# Supplementary material for: The presynaptic glycine transporter GlyT2 is regulated by the Hedgehog pathway in vitro and in vivo
Source: Commun Biol. 2021 Oct 18;4:1197. doi: 10.1038/s42003-021-02718-6 (PMC8523746; doi:10.1038/s42003-021-02718-6)
Supplement: Supplementary file 2 — Supplementary information [file 42003_2021_2718_MOESM2_ESM.pdf]

The presynaptic glycine transporter GlyT2 is regulated by the Hedgehog pathway in vitro and in vivo

Andrés de la Rocha-Muñoz[1,2,#], Enrique Núñez[1,2,#], Anjali Amrapali Vishwanath[3], Sergio Gómez-López[1], Dhanasak Dhanasobhon[3], Nelson Rebola[3], Beatriz López-Corcuera[1,2], Jaime de Juan-Sanz[3]* & Carmen Aragón[1,2]

[1]Centro de Biología Molecular "Severo Ochoa", Universidad Autónoma de Madrid, Consejo Superior de Investigaciones Científicas, 28049, Madrid, Spain. [2]IdiPAZ, Hospital Universitario La Paz, Madrid, Spain.[3]Sorbonne Université and Institut du Cerveau et de la Moelle Epinière (ICM) - Hôpital Pitié-Salpêtrière, Inserm, CNRS, Paris, France. [#]These authors contributed equally: A. de la Rocha-Muñoz and E. Núñez. *corresponding author: jaime.dejuansanz@icm-institute.org

Supplementary Information

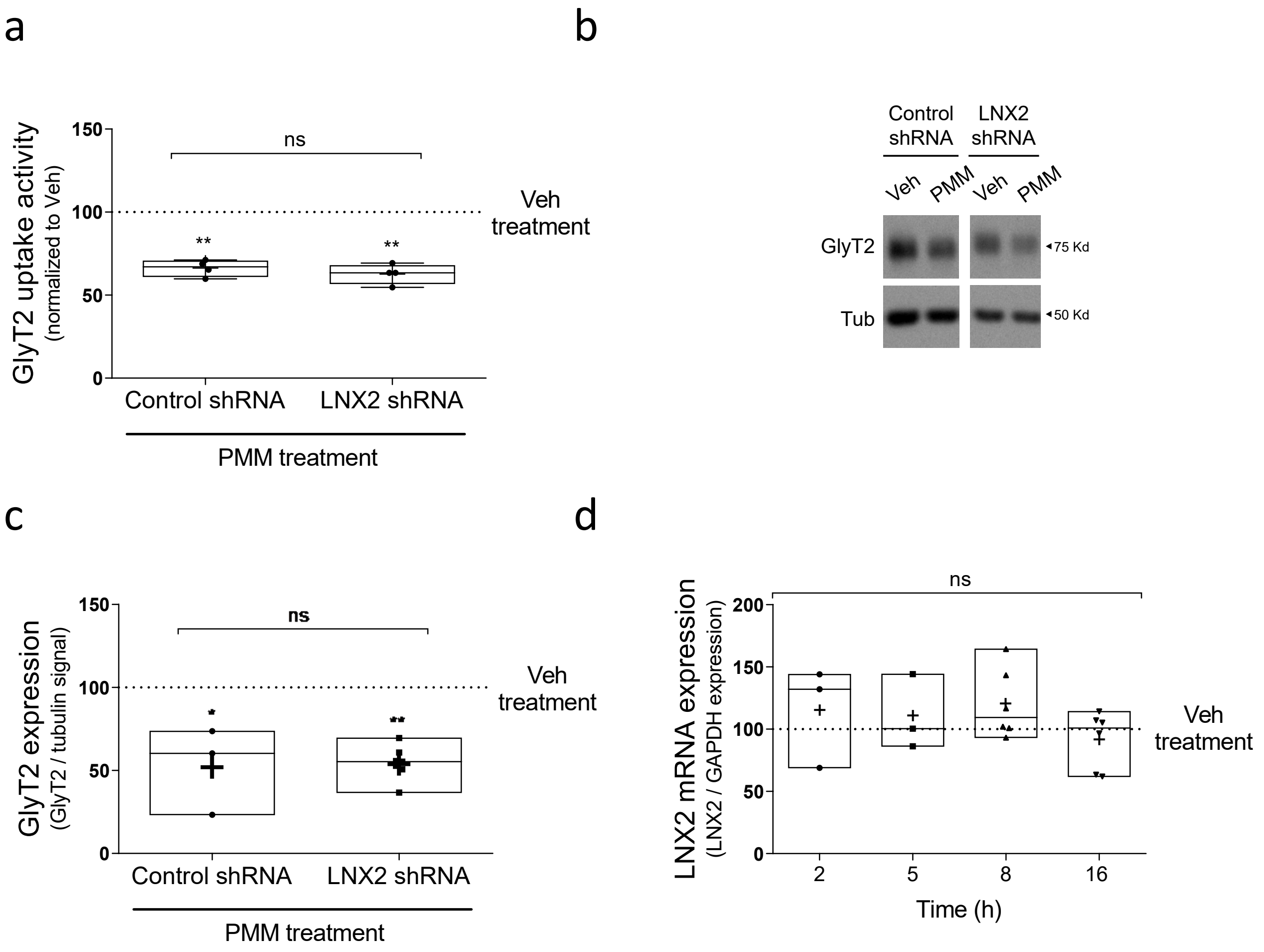

Fig. S1. The E3 ubiquitin ligase LNX2 does not appear to be involved in the ubiquitination of GlyT2 induced by purmorphamine.

a) Neuronal cultures infected with LNX2 shRNA or scrambled shRNA were treated with or without (vehicle) 10 µM PMM during 16 h and glycine transport rates were measured using [^{3}H]-glycine transport assays. Glycine transport shown is normalized against control conditions. **p (Control shRNA: Veh vs PMM) = 0.0037, **p (LNX2 shRNA: Veh vs PMM) = 0.0026; ^{ns}p (PMM: Control shRNA vs LNX2 shRNA) = 0.6814, using *Holm-Sidak's multiple comparisons test*, n = 4. **b)** Neuronal cultures infected with LNX2 shRNA or scrambled shRNA were treated with or without (vehicle) 10 µM PMM during 16 h and were subjected to western blot analysis using anti-GlyT2 antibodies. Tubulin was used as protein loading control. Note that ablation of LNX2 does not abolished the decrease in GlyT2 expression induced by treatment with PMM. **c)** Quantification of GlyT2 expression was normalized against tubulin. *p (Control shRNA: Veh vs PMM) = 0.0280, **p (LNX2 shRNA: Veh vs PMM) = 0.0023, ^{ns}p (PMM: Control shRNA vs LNX2 shRNA) = 0.9401, using *Holm-Sidak's multiple comparisons test*, n (Control shRNA: Veh & PMM) = 3, n (LNX2 shRNA: Veh & PMM) = 7. **d)** Neuronal cultures were treated with 10 µM purmorphamine at the times indicated. Total RNA was extracted from cells and quantification of GlyT2 mRNA was determined by qPCR. Relative mRNA levels of GlyT2 were determined by qPCR using Glyceraldehyde-3-phosphate dehydrogenase (GAPDH) as housekeeping gene (arbitrary units). n.s.= not significantly different, using *Kruskall-Wallis test*, n = 3. PMM: purmorphamine.

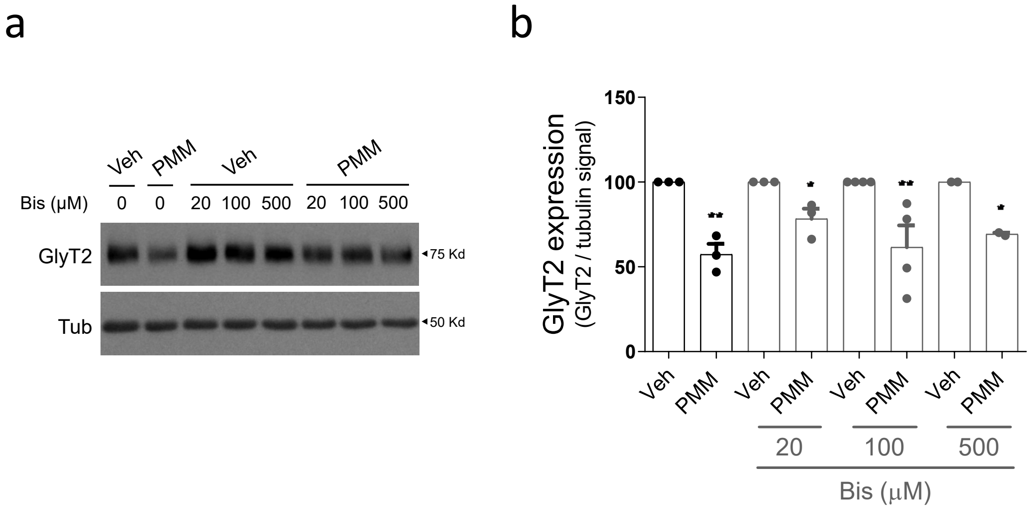

Fig. S2. Inhibition of PKC does not prevent the reduction of GlyT2 expression induced by purmorphamine.

a) Representative immunoblot of primary brainstem and spinal cord neurons. Cells were treated with 10 µM purmorphamine or vehicle for 16 h and without or with bisindoleylmaleimide at the indicated concentrations and the expression of GlyT2 was analyzed by immunobloting. Tubulin is used as protein loading control. **b)** Quantification of GlyT2 expression is normalized to the corrected signal against tubulin. **p (Control: Veh vs PMM) = 0.0020, *p (Bis 20 µM: Veh vs PMM) = 0.0491, **p (Bis 100 µM: Veh vs PMM) = 0.0018, *p (Bis 500 µM: Veh vs PMM) = 0.0491, using *Holm-Sidak's multiple comparisons test*, n (Control: Veh vs PMM) = 3, n (Bis 20 µM: Veh vs PMM) = 3, n (Bis 100 µM: Veh vs PMM) = 4, n (Bis 500 µM: Veh vs PMM) = 2. PMM: purmorphamine, Bis: bisindoleylmaleimide .

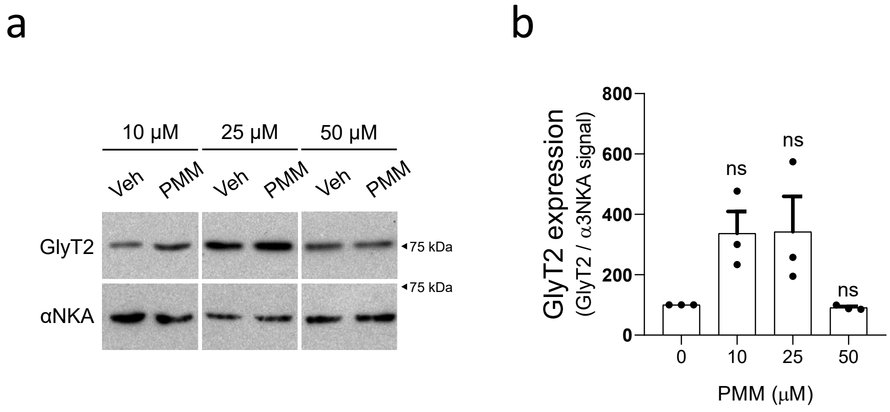

Fig. S3. Purmorphamine treatments do not show any conclusive effect on GlyT2 protein expression in zebrafish embryos.

a) Representative immunoblot of 48 hpf zebrafish embryos. 24 hpf embryos were incubated with 10, 25 and 50 µM purmorphamine or with vehicle alone for 24h (4 embryos per point) and the expression of GlyT2 was analyzed by immunoblotting. αNKA is used as protein loading control. **b)** Quantification of GlyT2 expression is normalized to the corrected signal against α3NKA. **p (Veh vs. PMM 10 µM) = 0.3312, **p (Veh vs. PMM 25 µM) = 0.4147, **p (Veh vs. PMM 50 µM) = 0.9143, using *Kolmogorov-Smirnov test*, n= 3. PMM: purmorphamine

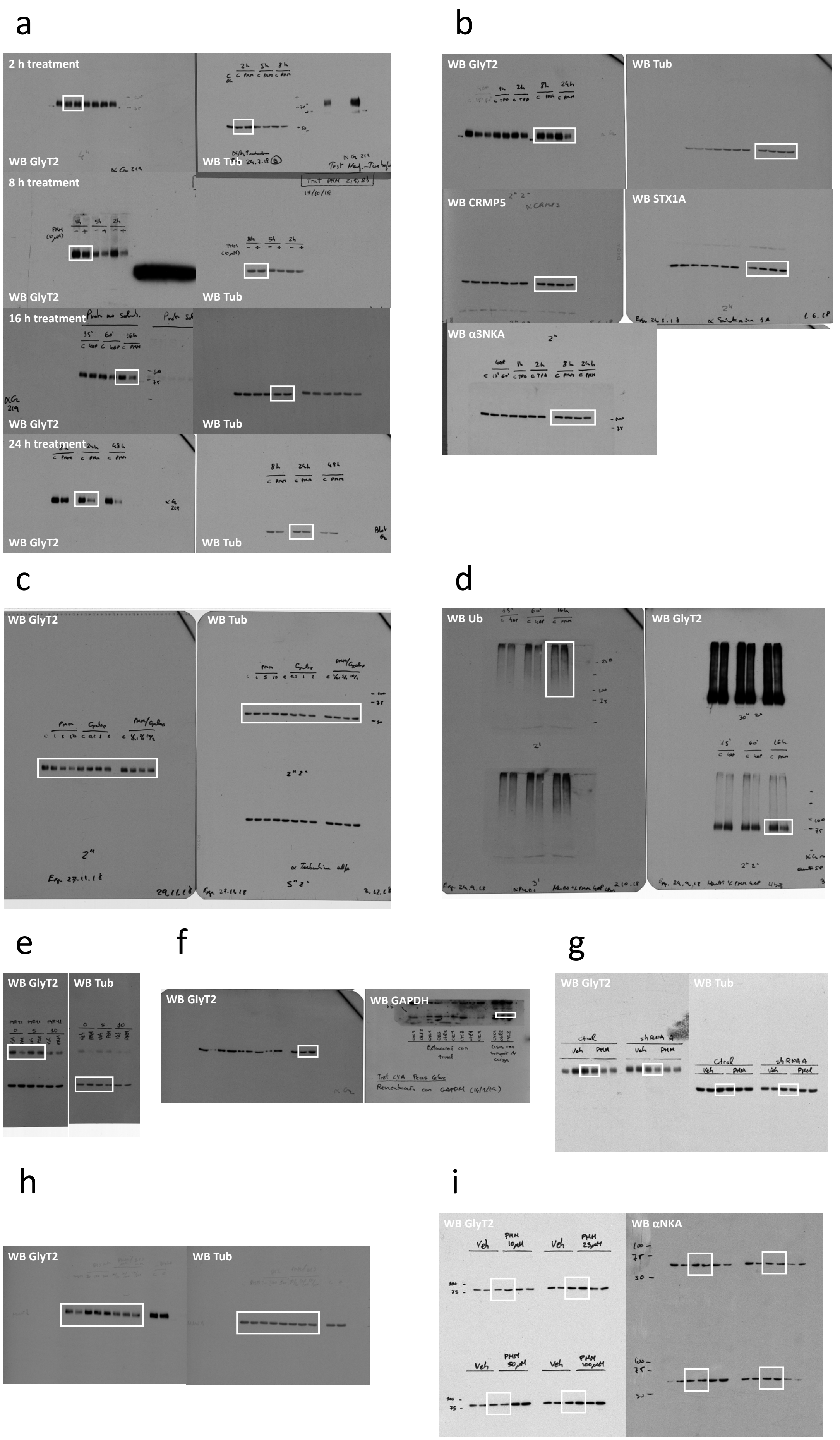

Fig. S4. Full scanned uncropped blots.

a) Uncropped blots Fig 1b. **b)** Uncropped blots Fig 1d. **c)** Uncropped blots Fig 2b. **d)** Uncropped blots Fig 3b. **e)** Uncropped blots Fig 3d. **f)** Uncropped blots Fig 4a. **g)** Uncropped blots Fig S1b. **h)** Uncropped blots Fig S2a. **i)** Uncropped blots Fig S3a.
