# Supplementary material for: The presynaptic glycine transporter GlyT2 is regulated by the Hedgehog pathway in vitro and in vivo
Source: Commun Biol. 2021 Oct 18;4:1197. doi: 10.1038/s42003-021-02718-6 (PMC8523746; doi:10.1038/s42003-021-02718-6)
Supplement: Supplementary file 3 — Description of Additional Supplementary Files [file 42003_2021_2718_MOESM3_ESM.pdf]

Description of Additional Supplementary Files

File name: Supplementary Data 1.
Description: Source data represented in the graphs of the main figures and supplementary figures of the article.
