# Supplementary material for: The presynaptic glycine transporter GlyT2 is regulated by the Hedgehog pathway in vitro and in vivo
Source: Commun Biol. 2021 Oct 18;4:1197. doi: 10.1038/s42003-021-02718-6 (PMC8523746; doi:10.1038/s42003-021-02718-6)
Supplement: Supplementary file 5 — Reporting Summary [file 42003_2021_2718_MOESM5_ESM.pdf]

nature research

Corresponding author(s): de Juan-Sanz, Jaime

Last updated by author(s): Sep 7, 2021

Reporting Summary

Nature Research wishes to improve the reproducibility of the work that we publish. This form provides structure for consistency and transparency in reporting. For further information on Nature Research policies, see our Editorial Policies and the Editorial Policy Checklist.

Statistics

For all statistical analyses, confirm that the following items are present in the figure legend, table legend, main text, or Methods section.

n/a	Confirmed	
☐	☒	The exact sample size (*n*) for each experimental group/condition, given as a discrete number and unit of measurement
☐	☒	A statement on whether measurements were taken from distinct samples or whether the same sample was measured repeatedly
☐	☒	The statistical test(s) used AND whether they are one- or two-sided<br>*Only common tests should be described solely by name; describe more complex techniques in the Methods section.*
☒	☐	A description of all covariates tested
☒	☐	A description of any assumptions or corrections, such as tests of normality and adjustment for multiple comparisons
☐	☒	A full description of the statistical parameters including central tendency (e.g. means) or other basic estimates (e.g. regression coefficient) AND variation (e.g. standard deviation) or associated estimates of uncertainty (e.g. confidence intervals)
☐	☒	For null hypothesis testing, the test statistic (e.g. *F*, *t*, *r*) with confidence intervals, effect sizes, degrees of freedom and *P* value noted<br>*Give P values as exact values whenever suitable.*
☒	☐	For Bayesian analysis, information on the choice of priors and Markov chain Monte Carlo settings
☒	☐	For hierarchical and complex designs, identification of the appropriate level for tests and full reporting of outcomes
☒	☐	Estimates of effect sizes (e.g. Cohen's *d*, Pearson's *r*), indicating how they were calculated

Our web collection on statistics for biologists contains articles on many of the points above.

Software and code

Policy information about availability of computer code

Data collection	Analysis of confocal images was performed using ImageJ software. Western blot bands were quantified using Image Lab Software (Bio-Rad).
Data analysis	All data and statistical analyses were performed using GraphPad Prism (GraphPad Software).

For manuscripts utilizing custom algorithms or software that are central to the research but not yet described in published literature, software must be made available to editors and reviewers. We strongly encourage code deposition in a community repository (e.g. GitHub). See the Nature Research guidelines for submitting code & software for further information.

Data

Policy information about availability of data

All manuscripts must include a data availability statement. This statement should provide the following information, where applicable:

- Accession codes, unique identifiers, or web links for publicly available datasets
- A list of figures that have associated raw data
- A description of any restrictions on data availability

The data generated and analyzed during this study are available from the corresponding author on reasonable request. We also show uncropped original blots and datasets corresponding to figure data in the Supplementary Material.

Field-specific reporting

Life sciences study design

All studies must disclose on these points even when the disclosure is negative.

Sample size	No sample size calculations were performed. Sample sizes were chosen based on established best practices in the field for the experimental methods used.
Data exclusions	No data were excluded from the analyses
Replication	Experimental findings were reliably reproduced. We did find variability in some of our observations, which we show by using box plots in the figures.
Randomization	Samples were organized into experimental groups according to their experimental condition. We did not perform experiments that require randomization. Controlling for covariates is not relevant because all experiments involved neurons from the same rat embryos or inbred zebrafish embryos, in which comparisons were between samples treated or not treated with pharmacological agents or shRNA vectors.
Blinding	Data acquisition was not blinded.

Reporting for specific materials, systems and methods

We require information from authors about some types of materials, experimental systems and methods used in many studies. Here, indicate whether each material, system or method listed is relevant to your study. If you are not sure if a list item applies to your research, read the appropriate section before selecting a response.

Materials & experimental systems

n/a	Involved in the study	
☐	☒	Antibodies
☒	☐	Eukaryotic cell lines
☒	☐	Palaeontology and archaeology
☐	☒	Animals and other organisms
☒	☐	Human research participants
☒	☐	Clinical data
☒	☐	Dual use research of concern

Methods

n/a	Involved in the study	
☒	☐	ChIP-seq
☒	☐	Flow cytometry
☒	☐	MRI-based neuroimaging

Antibodies

Antibodies used	Antibodies against N-terminus of GlyT2 and GlyT1 were generated in house, while the other primary antibodies used were: α3NKA (Santa Cruz Biotechnology, sc-16052), CRMP5 (Abcam, ab36203), Syntaxin 1A (Synaptic Systems), anti-ubiquitin (P4D1, Santa Cruz, sc-8017), anti-α-tubulin (T-6074, Sigma-Aldrich).
Validation	- GlyT2 antibody was generated in house and validated in this publication: Gomeza, J. et al (2003). Deletion of the mouse glycine transporter 2 results in a hyperekplexia phenotype and postnatal lethality. Neuron, 40(4), 797-806.<br>- GlyT1 antibody was generated in house and validated by not detecting a signal in mock COS7 cells but detecting a specific band when GlyT1 was transfected in these cells (data not shown).<br>- α3NKA (Santa Cruz Biotechnology, sc-16052) was validated by confirming a lost of signal when antibody was incubated with the Na+/K+-ATPase α3 antigen peptide (sc-365744 P).<br>- Other antibodies used were not validated by our group but showed unique bands corresponding to the expected molecular weights when analyzed in western blot, indicating their likelihood of detecting specifically their corresponding protein.

Animals and other organisms

Policy information about studies involving animals; ARRIVE guidelines recommended for reporting animal research

Laboratory animals	Animals used were Sprague-Drawley Rats and zebrafish (Danio rerio) embryos.
Wild animals	The study did not involve wild animals
Field-collected samples	The study did not involve samples collected from the field.
Ethics oversight	Zebrafish embryos and Wistar rats were bred under standard conditions at the Centro de Biología Molecular Severo Ochoa (CBMSO) in accordance with procedures approved in the Directive 2010/63/EU of the European Union with approval of the Research Ethics Committee of the Universidad Autónoma de Madrid (Comité de Ética de la Investigación UAM, CEI-UAM).

Note that full information on the approval of the study protocol must also be provided in the manuscript.
